# A Laissez-Faire Strategy Marked by Blinkers to Fulfill Established Pandemic Goals—The Case of Sweden

**DOI:** 10.3390/ijerph18189551

**Published:** 2021-09-10

**Authors:** Göran Svensson, Rocio Rodriguez

**Affiliations:** 1 School of Communication, Leadership and Marketing, Kristiania University College, 0107 Oslo, Norway; goran.svensson@kristiania.no; 2 Faculty of Economics and Business, University of Murcia, 30100 Murcia, Spain

**Keywords:** healthcare, health policy, public health pandemic, COVID-19

## Abstract

Objectives: To examine (i.e., contextualize and visualize) the consequences of a laissez-faire strategy characterized by blinkers to fulfill established pandemic goals. The aim is to shed light on the implementation of pandemic measures based on post hoc (after-the-fact) reactions and actions instead of pre hoc ones (in advance). Study Design: This study is based on weekly updates of pandemic variables (i.e., cases, tests, percentage of positive tests, hospitalizations, Intensive Care Unit (ICU) admissions, deceased, and 7- and 14-day incidence) in Sweden from the start of the pandemic in March 2020 until March 2021. Method: This study reports the empirical findings based on Swedish pandemic variables during 52 consecutive weeks, related to the pandemic, all of which has been divided into three time periods to separate the 1st and 2nd waves of the pandemic, and considers them all together as one time period. Results: The findings illustrate the implementation of pandemic measures and the subsequent consequences of a laissez-faire strategy characterized by blinkers. People become diseased and then deceased. This reveals strong associations between the assessed pandemic variables and its subsequent consequences on morbidity and mortality, based on post hoc reactions and actions. Conclusions: The implementation of a pandemic strategy should react and act pre hoc, and to take the safe with the unsafe. Governments and public health agencies should take into account the inevitable associations between pandemic variables. Intertwined pre hoc measures of prevention, enforcement, and monitoring should be implemented in society to avoid the implementation of a laissez-faire strategy based on post hoc reactions and actions.

## 1. Introduction

The research objective of this study is to examine (i.e., contextualize and visualize) the consequences of a laissez-faire strategy characterized by blinkers to fulfill established pandemic goals. The aim is to shed light on the implementation of pandemic measures based on post hoc (after-the-fact) reactions and actions instead of pre hoc ones (in advance). This study targets the implementation of a pandemic strategy by the government and the Agency of Public Health in Sweden.

The implementation of a pandemic strategy by the government and the Agency of Public Health in Sweden has stood out internationally and often in an unfavorable light [1,2]. It offers a contrasting point of reference and benchmark in relation to favorable implementations of pandemic strategies in the other Nordic countries [3].

In fact, the implementation of the pandemic strategy in Sweden has to a large extent been less strict and intrusive for the population than in many other countries [4,5], such as Denmark, Finland, and Norway, but also in comparison to most European countries. Sweden has not imposed confinement and compulsory measures on the population as done in Italy, France, and Spain, amongst others. Furthermore, Sweden has not closed its borders during the pandemic as done in Denmark, Finland, and Norway [3].

The absence of strict and compulsory measures to protect the population indicates an implementation of the pandemic strategy in Sweden with an air of laissez-faire, with the post hoc effects and consequences of exposing the population to severe morbidity and mortality appearing to have been downgraded, underestimated, or, at worst, neglected.

In fact, the mortality rate of Sweden is among the highest in the world [6,7], despite the country having a highly developed economy with a small population with low density [8]. It is without doubt the Nordic country worst affected by pandemic-related morbidity and mortality [6,7].

The social and cultural contexts of Denmark, Finland, and Norway resemble Sweden to a large extent [9,10,11]. However, the governments and agencies of public health in the neighboring Nordic countries have reacted and acted differently with strict, compulsory, and pre hoc measures to handle the pandemic to protect their respective populations. Sweden has mainly reacted in the opposite manner and acted with voluntary recommendations and post hoc measures for the population. The neighboring Nordic countries appear to have handled the pandemic as a societal crisis, while Sweden appears to regard it as a health crisis [3,12]. The governments of the neighboring Nordic countries have joined societal forces, competences, and skills, taking responsibility for imposing compulsory restrictions and asking for people’s understanding and collaboration.

There is also another difference compared to the neighboring Nordic countries, namely that the Swedish government and the Agency of Public Health to a larger extent transferred the responsibility for reacting and acting to the population using moral suasion, rather than imposing strict and compulsory measures to control the pandemic and its societal consequences, as done in Denmark, Finland, and Norway. The Government Offices [13] writes explicitly: “…every person in Sweden needs to take responsibility. By everyone, we can keep the level of infection spread down…”. Consequently, the Swedish government holds the population accountable, rather than accepting its own political and societal responsibility to protect the population.

## 2. Study Design

This study is based on weekly updates of a set of pandemic variables in Sweden from the start of the pandemic in March 2020 until March 2021. The data were gathered from the Agency of Public Health [14] (2020–2021), the National Board of Health and Welfare [15] (2020–2021), and the Swedish Intensive Care Registry—SIR [16] (2020–2021).

## 3. Method

This study reports the empirical findings based on Swedish pandemic variables during 52 consecutive weeks, related to the pandemic, all of which has been divided into three time periods to separate the 1st and 2nd waves of the pandemic, and considers them all together as one time period.

The criterion for determining the end of the 1st wave and the beginning of the 2nd is based on when the percentage of positive tests reached its lowest level (i.e., 1.1%) since the inception of the pandemic. The 1st wave therefore ended at the end of August (i.e., week number 36) and the beginning of September (i.e., week number 37), as shown in Table 1.

A series of statistical analyses and a graphical illustration are reported in the next section based on three time periods: (i) from March 2020 to August 2020—1st wave; (ii) from September 2020 to March 2021—2nd wave; and (iii) from March 2020 to March 2021—1st and 2nd waves.

## 4. Results

### 4.1. Graphical Illustration

This study focuses on eight pandemic variables and their average count per day on a weekly basis, namely: (i) cases, (ii) tests, (iii) percentage of positive tests, (iv) hospitalizations, (v) ICU (Intensive Care Unit) admissions, (vi) deceased, (vii) 7-day incidence per 100,000 people, and (viii) 14-day incidence per 100,000 people.

**Figure 1:**
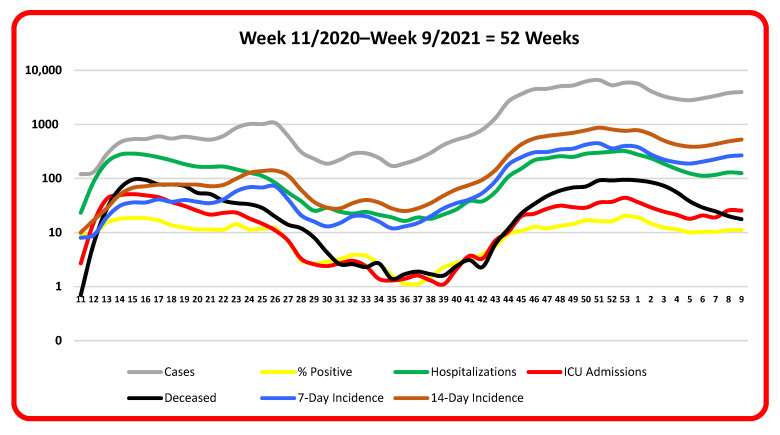
Pandemic variables through time across 1st and 2nd waves.

Figure 1 reports the assessed pandemic variables through time (i.e., longitudinal observation) and their connectedness across the 1st and 2nd waves. It is striking how the assessed pandemic variables follow a wave pattern since the pandemic started to the present. It should be noted that Figure 1 excludes the number of tests, due to comparatively much larger numbers than the other pandemic variables that would flatten the wave pattern.

The coherent wave patterns in Figure 1 show that there are strong associations between the assessed pandemic variables. The raw data of Figure 1 are depicted in Table 1 and the statistical correlations are subsequently reported in Table 2. Nevertheless, the decline in deceased (i.e., bold black line) around weeks four and five in 2021 shows the effect of vaccinations undertaken among the elderly and fragile population.

Table 1 reports the underlying statistics of each pandemic variable during 52 weeks from week number 11 in 2020 until week number 9 in 2021. The table reports the univariate statistics based on the average count per day on a weekly basis across each pandemic variable. Consequently, a year of variable statistics is summarized to visualize the underlying wave pattern of raw data as displayed in Figure 1.

The upper part of Table 1 provides three main headings: (i) week number; (ii) average count per day on a weekly basis regarding: number of confirmed cases, percentage of positive tests, number of hospitalizations, number of ICU admissions, and number of deceased, and finally (iii) incidence per 100,000 people based on time periods of seven and fourteen days.

The lower part of Table 1 displays three time periods: (i) 52 weeks from week 11 (9 March 2020) to week 09 (1 March 2021); (ii) 26 weeks from week 11 (9 March 2020) to week 37 (9 September); and (iii) 26 weeks from week 37 (9 September 2020) to week 9 (1 March 2021).

A visual inspection of Table 1 reveals that the assessed pandemic variables increase and decrease in a similar wave pattern as shown in Figure 1 throughout the time period of 52 weeks. Logically, when confirmed cases increase, the 7- and 14-day incidences increase as well, which also applies to the number of tests.

However, when it comes to the percentage of positive tests, it appears that the hospitalizations and ICU admissions follow the ups and downs of this pandemic variable to a larger extent, and the numbers of deceased follow the ups and downs of the percentage of positive tests to a larger extent as well.

Table 1 also shows (lower part) that the average counts across the assessed pandemic variables in the 1st wave are generally lower than the 2nd wave. On the one hand, the numbers of tests were much lower in the 1st wave, and therefore generate much lower numbers of confirmed cases and 7- and 14-day incidences. On the other hand, the average percentage of positive tests is similar between the 1st and 2nd waves, and the differences between hospitalizations and ICU admissions and deceased are also equal.

### 4.2. Bivariate Statistics across Time Periods

Consequently, the statistics of pandemic variables reported in Table 1 may be divided into two groups of interconnected pandemic variables consisting of: (i) cases, tests, and the 7- and 14-day incidences; and (ii) percentage of positive tests, hospitalizations, ICU admissions, and deceased. Revealing the underlying pattern in Figure 1 and Table 1, Table 2 therefore reports the statistical correlations between the assessed pandemic variables.

Consequently, Table 2 reports multiple correlation tests (Pearson) across three time periods: (i) 52 weeks from week 11 (9 March 2020) to week (1 March 2021); (ii) 26 weeks from week 11 (9 March 2020) to week 37 (9 September); and (iii) 26 weeks from week 37 (9 September 2020) to week 9 (1 March 2021).

Table 2 indicates that some of the reported statistical correlations are more relevant to each other than others, namely: (i) cases, tests, the 7- and 14-day incidences; and (ii) percentage of positive tests, hospitalizations, ICU admissions, and deceased. We therefore discuss the statistics reported within each of these two groups of pandemic variables.

#### 4.2.1. Cases, Tests, and the Incidence of 7- and 14-Days

As shown in Table 2, the average count per day across 52 weeks between cases on the one hand, and 7- and 14-day incidence on the other, demonstrated that the correlations are all significant at *p* = 0.000 with coefficients of 0.994–1.000. Similarly, the correlations between tests and 7- and 14-day incidence are also all significant at *p* = 0.000, with regression coefficients of 0.864–0.878.

However, there is a major difference between tests and the 7- and 14-day incidence between the 1st and 2nd waves. The tests in the 1st wave were, as pointed out previously, very few in number, providing non-significant correlations with *p*-values ranging from 0.596–0.997, and coefficients ranging from 0.001–0.109. By contrast, the correlations between tests and the 7- and 14-day incidence in the 2nd wave are significant with *p*-values at 0.000, and coefficients ranging from 0.854–891. Consequently, the correlations between the pandemic variables within this group are significant.

#### 4.2.2. Percentage of Positive Tests, Hospitalizations, ICU Admissions, and Deceased

Table 2 shows that with the average count per day across 52 weeks between the percentage of positive tests on the one hand, and hospitalizations, ICU admissions, and deceased on the other, the correlations are all significant at *p* = 0.000 with coefficients of 0.874–0.937. In fact, the correlations between the percentage of positive tests, hospitalizations, ICU admissions, and deceased are also all significant at *p* = 0.000, with coefficients of 0.874–0.942.

The correlations between the same pandemic variables are all significant at *p* = 0.000 in the 1st wave (i.e., week 11–36) with coefficients of 0.847–0.989. Similarly, the correlations are also all significant at *p* = 0.000, with coefficients of 0.902–0.970 in the 2nd wave (i.e., week 37–09). Consequently, the correlations between the pandemic variables within this group are also significant.

## 5. Discussion

A closer look at the statistics across a range of pandemic variables between Sweden and the neighboring Nordic countries shows substantial differences [6,7], such as: (i) the number of confirmed cases is 3.5 times higher per capita in Sweden than in Denmark, Finland, and Norway averaged together; (ii) the number of performed tests per capita in the neighboring countries together is 9 times higher than Sweden; and (iii) death tolls are 5.5 times higher per capita in Sweden. In addition, the death tolls per million inhabitants are [6,7]: (i) Sweden, 1400; (ii) Denmark, 430; (iii) Finland, 167; and (iv) Norway, 141.

The 14-day incidence per 100,000 people has been extremely high in Sweden (on average, 600+) for the last six months, while the neighboring Nordic countries have managed to continuously keep their incidence much lower [6]. Consequently, the outcome of the post hoc reactions and actions regarding implementing the pandemic strategy by the Swedish government and the Agency of Public Health stand out drastically and negatively [6] in comparison to the often pre hoc ones of the neighboring Nordic countries, although they are all economically, socially, environmentally, and culturally similar [9,10,11].

A crucial issue is that the implementation of the pandemic strategy by the Swedish government and the Agency of Public Health appears to have neglected the formulated goals. The goals formulated by the Government Offices [13] “to reduce the rate of the spread of infection, i.e., to flatten the curve so that not many people become ill at the same time” has been taken into consideration somewhat questionably by the Swedish government.

The reported findings show that the numbers of morbidity and mortality have been much higher in Sweden, in comparison to Denmark, Finland, and Norway, so the strategic goals established cannot really by regarded as accomplished. The goals formulated by the Agency of Public Health [14] “to minimize morbidity and mortality in the population” and “minimize other negative consequences for the individual and society” appear to have been largely neglected. Our findings show that the Agency of Public Health appears not to have attempted, and tentatively not even considered, minimizing the morbidity and mortality in the population. By contrast, the neighboring Nordic countries have indeed striven to minimize them. Furthermore, it is questionable whether the negative consequences for the individual and society have been minimized; at best they have been reduced to a minor extent.

We therefore argue that the pandemic strategy goals formulated by the Swedish government and the Agency of Public Health have not been properly addressed and therefore not accomplished. Consequently, the implementation of the pandemic strategy has been marked by blinkers, basically neglecting the formulated goals. The outcome of the pandemic strategy implemented by the Swedish government and the Agency of Public Health has failed in comparison to the more successful ones of Denmark, Finland, and Norway in terms of morbidity and mortality. In addition, the negative impact of the pandemic on the GDP growth of Denmark [17] (−2.7%), Finland [18] (−2.8%), Norway [19] (−2.5%), and Sweden [20] (−2.8%) was about the same, though Sweden kept society more open than the other Nordic countries.

We contend that the strategy applied by the Swedish government and the Agency of Public Health did not acknowledge and handle CoV-SARS-2 (i.e., COVID-19) as a pandemic virus, but approached it as an influenza virus. The strategy applied during the 1st wave was therefore characterized by open borders, minimal testing, voluntary home quarantines, and insignificant infection tracing, as well as no recommendations or compulsory measures for using face masks (e.g., medical face masks, cloth face coverings, FFP2, or FFP3) on public transport and in indoor public spaces. In fact, hardly any strict measures were implemented during the 1st wave by the Swedish government and the Agency of Public Health.

The strategy applied in the 1st wave appeared to strive towards herd immunity in the population, although no empirical evidence existed of its feasibility at the time. We contend that the spread of infection in the population was a deliberate strategic choice, although the Swedish government and Agency of Public Health fiercely deny this officially. If this was indeed the choice they made, it was a reckless strategic one that caused unnecessary morbidity and mortality in the population. Furthermore, the pandemic numbers of morbidity and mortality in comparison to the neighboring Nordic countries speak for themselves [6,7].

The Swedish government and Agency of Public Health also deny that the strategy has changed during the pandemic. However, it is quite evident that the strategy changed after the 1st wave that turned out to be much worse in terms of morbidity and mortality than in the neighboring Nordic countries [6,7]. The Swedish government and the Agency of Public Health were obliged to enforce stricter measures to protect the population in the 2nd wave, as the rate of infection again began to rocket and cause unsustainable levels of morbidity and subsequent elevated rates of mortality. However, the statistics of pandemic variables reported in Table 1 consistently demonstrate that the measures implemented in the 2nd wave were still too lax and insufficient, as well as most likely implemented too late. The table illustrates the post hoc reactions and actions undertaken by the Swedish government and the Agency of Public Health. It is a strategy characterized by narrow vision and a lack of insight—blinkers.

Subsequently, the Swedish government and the Agency of Public Health reacted and acted differently in the 2nd wave, but massive morbidity and mortality still followed in the population. The pandemic outcome in Sweden can thus indeed be described as the result of an inappropriate laissez-faire strategy. The implementation of pandemic measures can be described as based on post hoc reactions and actions rather than pre hoc and timely measures. 

## 6. Conclusions

A series of lessons that serve as recommendations for the future can be derived from the Swedish laissez-faire strategy. The Swedish strategy is characterized by blinkers rather than openness. The inertia constraining reactions and actions in handling the pandemic is clearly associated with elevated morbidity and mortality. The implementation of the strategy by the Swedish government and the Agency of Public Health has failed to confront the lack of scientific knowledge, experiences, and measures undertaken in other countries (e.g., Denmark, Finland, and Norway). The strategy has also suffered from a lack of preparation and precautions that are so urgently needed to confront the health effects of pandemic morbidity and mortality. It is also characterized by insufficient monitoring and control of the spread of infection, rather than pre-determined protocols and extensive follow-up procedures in the population. The strategy has also been based mostly on voluntary measures rather than compulsory ones. The Swedish strategy has neglected the logic of better to be safe than sorry.

A series of lessons serving as recommendations for the future can also be outlined based on the implementation of the pandemic measures that has been based on post hoc reactions and actions, rather than pre hoc (or at least timely) ones. The Swedish government and the Agency of Public Health have been reluctant to undertake preventive measures and pre hoc decision making, and instead mostly reacted and acted with tardiness and simply insufficiently. It also appears that the measures have not been properly aligned with the actual developments in society. The goals established by the Swedish government and the Agency of Public Health have been simply ignored by much of the broader public, which, not surprisingly, questions the logic and appropriateness of the reactions and actions undertaken. The Swedish implementation has neglected to take actions just in case, let alone just in time.

The blinkers and post hoc reactions and actions characterizing the Swedish laissez-faire strategy and implementation of pandemic measures raise various ethical and moral concerns. This scenario has caused far higher morbidity and mortality than in the neighboring Nordic countries. Historically, Sweden is normally widely known internationally for reacting and acting to protect the well-being of the population, but the pandemic reveals an ethical and moral decay in the strategy and implementation of pandemic measures by the Swedish government and the Agency of Public Health.

The Swedish laissez-faire strategy and the implementation of pandemic measure-based post hoc reactions and actions have therefore most likely unnecessarily caused immense human suffering and escalating death tolls in the population, compared to Denmark, Finland, and Norway.

We conclude that the Swedish government and the Agency of Public Health have, since the inception of the pandemic, mostly refused to enforce strict and compulsory measures to protect the population. They have neglected their own goals for combating the pandemic. Furthermore, the Swedish government and the Agency of Public Health reacted and acted weakly in comparison to the neighboring Nordic countries, and reacted and acted differently to most European countries. 

The pandemic variables assessed and reported in this study provide evidence that the post hoc implementation by the Swedish government and the Agency of Public Health has failed to fulfill its goals. The core evidence is the numbers of the pandemic variables which speak for themselves.

We conclude that the implementation of the pandemic strategy in Sweden has failed, in comparison to the neighboring Nordic countries, that have all succeeded in keeping infection, morbidity, and mortality rates much lower.

The pandemic variables of morbidity and mortality assessed in this study reveal that cases and percentages of positive tests cannot be allowed to rise beyond control. On the contrary, contemporary experiences across countries (e.g., Australia, China, South Korea, Taiwan, and New Zealand) clearly demonstrate that the pandemic can be handled and kept under control with very strict and compulsory measures to protect the population (e.g., as also done excellently in Finland and Norway, and also well in Denmark).

We therefore contend that the implementation of a pandemic strategy should react and act rapidly and firmly. Reacting and acting with tardiness and indulgence extracts a high price in terms of infection, morbidity, and mortality rates in society. The increase in cases and high percentages of positive tests lead to large numbers of disease cases (i.e., hospitalizations and ICU admissions) in the end.

This study therefore contributes to contextualizing and visualizing the severe consequences of neglecting the strong associations between the assessed pandemic variables. The reported findings provide a foundation and justification for establishing and outlining pre hoc measures of implementation rather than post hoc ones. The findings also underline the importance of implementing intertwined measures of prevention, enforcement, and monitoring in society. Finally, this study illustrates the consequences of implementation based on a laissez-faire strategy caused by post hoc reactions and actions. The outcome is unavoidable; people become diseased and deceased.

## Figures and Tables

**Table 1:** Longitudinal statistics of pandemic variables.

Week	Average Count per Day						Incidence per 100,000	
	Cases	Tests	% Positive	Hospitalizations	ICU Admissions	Deceased	7 Days	14 Days
11	120	1284.3	9.3	23.3	2.7	0.7	8	10
12	132	1486.3	8.8	87.1	15.3	6.3	9	17
13	280	1764.1	15.7	197.6	41.7	27.1	19	28
14	461	2540.4	18.1	274.7	49.0	64.4	31	50
15	534	2840.0	18.7	288.3	51.4	94.9	36	67
16	536	2890.4	18.5	274.1	48.7	93.9	36	72
17	600	3508.6	17.0	247.0	45.0	78.0	41	77
18	547	4114.6	13.6	215.7	36.6	77.6	37	78
19	593	4161.1	12.3	184.7	31.1	72.9	40	77
20	553	4714.7	11.4	165.7	25.3	54.3	37	77
21	523	4140.9	11.4	163.4	21.5	51.4	35	72
22	611	5209.4	11.2	166.1	23.1	38.9	41	76
23	860	7023.1	14.6	147.7	23.4	35.0	58	99
24	1018	8668.3	11.3	128.3	18.3	33.4	69	127
25	1010	8829.0	11.8	111.9	14.6	28.6	68	137
26	1065	10,582.3	12.3	83.3	11.0	19.7	72	140
27	611	11,401.6	7.0	55.1	7.1	14.0	41	113
28	311	11,685.9	3.0	38.1	3.3	12.0	21	62
29	232	9913.3	2.6	25.4	2.6	8.1	16	37
30	188	8449.0	2.9	28.6	2.4	4.3	13	29
31	222	7565.6	3.2	24.3	2.7	2.6	15	28
32	288	7674.4	3.9	22.4	3.0	2.6	20	35
33	293	8089.6	3.8	24.1	2.4	2.3	20	40
34	241	9363.7	2.7	21.3	1.4	2.7	16	36
35	171	12,151.4	1.6	19.1	1.3	1.4	12	28
36	190	18,031.3	1.1	16.4	1.4	1.7	13	25
37	227	20,381.9	1.1	19.0	1.6	1.9	15	28
38	297	19,924.4	1.5	18.0	1.3	1.7	20	35
39	417	18,407.4	2.3	21.6	1.1	1.6	28	48
40	520	18,269.1	2.8	26.9	2.1	2.4	35	63
41	611	19,554.7	3.1	38.3	3.7	3.1	41	76
42	803	21,181.0	3.9	37.9	3.3	2.3	54	95
43	1308	23,534.6	5.6	56.3	7.1	6.1	89	143
44	2639	27,043.0	9.7	107.6	10.3	11.7	179	268
45	3629	32,574.7	10.7	152.1	20.0	22.4	246	425
46	4474	36,327.9	12.9	217.3	22.3	33.6	303	549
47	4563	37,244.3	11.9	237.0	27.6	47.4	309	612
48	5080	39,387.4	13.3	258.0	31.4	59.6	344	653
49	5262	37,318.4	14.4	250.3	29.4	68.0	357	701
50	6233	38,706.3	17.1	287.6	28.9	71.4	422	779
51	6597	41,061.1	16.3	299.7	35.9	92.1	447	869
52	5305	33,159.1	16.1	312.0	37.1	91.9	360	807
53	5904	28,715.9	20.7	321.6	44.0	94.7	344	760
1	5637	29,633.1	19.2	276.7	36.6	92.0	381	780
2	4136	28,692.0	14.7	236.6	29.3	85.1	362	659
3	3320	27,598.3	12.3	187.4	24.4	73.3	312	503
4	2953	26,071.0	11.5	149.4	21.3	55.6	260	423
5	2802	28,221.6	10.0	124.3	18.0	37.9	245	388
6	3041	29,908.9	10.3	112.3	20.7	29.1	226	394
7	3375	31,787.4	10.3	117.0	19.1	24.6	213	433
8	3815	34,088.3	11.1	129.6	25.7	20.1	224	485
9	3969	35,573.6	11.2	125.7	25.4	17.7	233	525
**Week**	**Cases**	**Tests**	**% Positive**	**Hospitalizations**	**ICU Admissions**	**Deceased**	**7 Days**	**14 Days**
11–09	1156.8	18,124.0	10.0	137.6	18.4	35.7	129	253
11–36	468.8	6849.4	9.5	116.7	16.4	31.9	32	63
37–09	1844.8	29,398.7	10.5	158.7	20.3	39.6	226	442

Note: ICU—Intensive Care Unit.

**Table 2:** Correlations and significances between pandemic variables.

Pearson Correlation Tests—Week 11–09/11–36/37–09								
Variable		Tests	% Positive	Hospitalizations	ICU Admissions	Deceased	7-Day Incidence	14-Day Incidence
**Cases**	Coefficient	0.88/−0.00/0.89	0.56/0.55/0.96	0.66/0.42/0.96	0.46/0.34/0.96	0.55/0.43/0.86	1.00/1.00/1.00	0.99/0.97/0.99
	Significance	0.00/0.99/0.00	0.00/0.00/0.00	0.00/0.03/0.00	0.00/0.09/0.00	0.00/0.03/0.00	0.00/0.00/0.00	0.00/0.00/0.00
	N	52/26/26	52/26/26	52/26/26	52/26/26	52/26/26	52/26/26	52/26/26
**Tests**	Coefficient		0.17/−0.71/0.76	0.31/−0.66/0.79	0.11/−0.68/0.79	0.20/−0.56/0.59	0.88/0.00/0.89	0.86/0.11/0.85
	Significance		0.23/0.00/0.00	0.02/0.00/0.00	0.46/0.00/0.00	0.16/0.00/0.00	0.00/0.99/0.00	0.00/0.60/0.00
	N		52/26/26	52/26/26	52/26/26	52/26/26	52/26/26	52/26/26
**% Positive**	Coefficient			0.94/0.94/0.95	0.94/0.93/0.97	0.87/0.85/0.90	0.56/0.55/0.96	0.56/0.44/0.97
	Significance			0.00/0.00/0.00	0.00/0.00/0.00	0.00/0.00/0.00	0.00/0.00/0.00	0.00/0.03/0.00
	N			52/26/26	52/26/26	52/26/26	52/26/26	52/26/26
**Hospitalizations**	Coefficient				0.94/0.99/0.95	0.94/0.95/0.94	0.66/0.42/0.96	0.67/0.33/0.97
	Significance				0.00/0.00/0.00	0.00/0.00/0.00	0.00/0.03/0.00	0.00/0.10/0.00
	N				52/26/26	52/26/26	52/26/26	52/26/26
**ICU Admissions**	Coefficient					0.89/0.92/0.90	0.46/0.34/0.96	0.46/0.24/0.98
	Significance					0.00/0.00/0.00	0.00/0.10/0.00	0.00/0.23/0.00
	N					52/26/26	52/26/26	52/26/26
**Deceased**	Coefficient						0.55/0.43/0.86	0.58/0.38/0.91
	Significance						0.00/0.03/0.00	0.00/0.06/0.00
	N						52/26/26	52/26/26
**7-Day** **Incidence**	Coefficient							0.99/0.97/0.99
	Significance							0.00/0.00/0.00
	N							52/26/26

## Data Availability

Public data available at the Agency of Public Health [14] (2020–2021), the National Board of Health and Welfare [15] (2020–2021), and the Swedish Intensive Care Registry—SIR [16] (2020–2021).
